# Does data protection law in South Africa apply to pseudonymised data?

**DOI:** 10.3389/fphar.2023.1238749

**Published:** 2023-11-23

**Authors:** Donrich Thaldar

**Affiliations:** ^1^ School of Law, University of KwaZulu-Natal, Durban, South Africa; ^2^ Petrie-Flom Center for Health Law Policy, Biotechnology and Bioethics, Harvard Law School, Cambridge, MA, United States

**Keywords:** code of conduct, data protection, POPIA, pseudonymisation, research, South Africa, transfer

## Abstract

The use of pseudonymised datasets is increasingly commonplace as research institutions seek to balance data utility with data security. Yet, a crucial question arises: How does South Africa’s Protection of Personal Information Act (POPIA) govern these datasets, especially given their ambiguous state between de-identification and possible re-identification? A thorough examination of POPIA suggests that the determination of whether a pseudonymised dataset is personal information—and thus whether processing the dataset falls within POPIA’s purview—must be informed by the *specific context* of the responsible party in possession of the pseudonymised dataset. When a research institution retains both the pseudonymised dataset and its linking dataset, the pseudonymised dataset remains identifiable and is thus personal information that falls within POPIA’s purview. However, when only the pseudonymised dataset—without the linking dataset—is transferred to another entity, it is non-personal information in the hands of such a recipient, thus freeing the recipient from POPIA compliance. Such a delineation offers research institutions greater flexibility in sharing and using pseudonymised datasets. Importantly, because the original provider of the pseudonymised dataset (who has the means to re-identify the dataset) remains governed by POPIA, the privacy rights of data subjects are not undermined.

## 1 Introduction

When sharing health research data, it is a legal and ethical imperative to secure any information that can identify *research participants*—or *data subjects* in privacy law terminology. A common technique used to accomplish this is to replace data subjects’ identifying information in the dataset to be used for research—and for sharing with collaborators—with unique codes. This is done while keeping another dataset that links these data subjects’ identifying information with their allocated codes. This technique—commonly referred to as *pseudonymisation*—attains non-identifiability of data subjects in certain *specific contexts*, viz where researchers have access to only the pseudonymised dataset and not to the linking dataset. However, pseudonymisation does not attain non-identifiability of data subjects in any or all contexts, as the linking dataset still exists and can be used by someone—perhaps now or in the future—to identify the data subjects in the pseudonymised dataset.

Since the identifiability of data subjects in a dataset is the fulcrum of determining whether statutory data protection law rules apply to such a dataset, it is important to know whether *context* is legally relevant when working with pseudonymised datasets. However, this has been controversial—so much so that this has already led to litigation in the European Union. In the recent case of *Single Resolution Board v European Data Protection Supervisor* (2023), the European Data Protection Supervisor adopted a context-agnostic stance that focused on the fact that when a dataset is pseudonymised the data subjects remain identifiable because someone, somewhere, still has the linking dataset that can be used to identify the data subjects. However, aligned with previous case law (*Breyer v Federal Republic of Germany*, 2016), the General Court of the EU decided against the European Data Protection Supervisor and held that the identifiability of data subjects must be determined based on the *specific context* of the relevant party before the court. The European Data Protection Supervisor filed an appeal against this judgment (European Data Protection Supervisor, 2023). The appeal will be heard by the Court of Justice of the EU.

This ongoing litigation in the EU raises the pertinent question: What would be the position in South Africa? The South African Protection of Personal Information Act (POPIA) (Protection of Personal Information Act 4, 2013) does not explicitly deal with pseudonymisation. Also, there is no South African case law on the topic, nor any guidance by the country’s Information Regulator. In this article, I analyse POPIA and propose that the South African position is that the identifiability of data subjects must be determined based on *specific context*.

## 2 Interpreting POPIA

I plot two complementary interpretative avenues through POPIA: The first focuses on the definitions of the terms “de-identify” and “re-identify” used in the exclusions clause—in particular, the phrase “reasonably foreseeable method” contained in these definitions—and interpret this reasonability standard by borrowing established legal principles from other branches of the law. The second interpretative avenue uses POPIA’s application clause as a point of departure and then analyses POPIA’s research exception. As will become evident, both of these interpretative avenues reach the same destination.

### 2.1 The exclusions clause and the definitions

POPIA’s exclusions clause, Section 6, provides that POPIA does not apply to the processing of personal information that has been de-identified to the extent that it cannot be re-identified again. However, it is not clear from this clause *who* must not be able to re-identify the de-identified information: Nobody in the entire world, or the specific responsible party in possession of the information?

To help find an answer to this question, the definitions of the terms “de-identify” and “re-identify” (in Section 1 of POPIA) should be considered. Both definitions use much of the same language and are mirror images of each other. They both relate to information that (a) identifies the data subject; (b) can be used or manipulated by a *reasonably foreseeable method* to identify the data subject; or (c) can be linked by a *reasonably foreseeable method* to other information that identifies the data subject. The difference is that de-identification is the *deletion* of such information [meaning information of type (a), (b), or (c)], while re-identification is the *resurrection* of such information that has been deleted. Note POPIA’s use of the phrase a “reasonably foreseeable method”*.* The concept of reasonableness is not unique to informational privacy law (as codified in POPIA) and is regularly used in other branches of South African law—especially the law of delict and administrative law. In these branches of the law, reasonableness is understood to entail an *objective* inquiry (*Cape Town Municipality v Bakkerud*, 1997; *Medirite Ltd v South African Pharmacy Council*, 2013). There is no reason to believe that the same would not also apply to POPIA. An objective inquiry means that when considering whether a pseudonymised dataset is in fact de-identified, and also when considering whether such a pseudonymised dataset can be re-identified, the test is not whether the responsible party *subjectively* foresaw a method that would re-identify the information, but rather whether a reasonable person—an abstraction—would have foreseen a method that would re-identify the information.

Although it is important that the definitions of “de-identify” and “re-identify” require an objective inquiry, this still does not solve the problem of the legal relevance of *context*. Must the reasonable person be conceived of in a context-agnostic way, or conceived of in a specific context? The well-established position in South African law of delict is to conceive of the reasonable person *in the position* of the person whose conduct is considered (*Mukheiber v Raath,* 1999). In other words, the objective inquiry is not context-agnostic, but firmly anchored in a specific context. Therefore, if applied to POPIA, the objective test is not whether a reasonable person *anywhere in the world* would have foreseen a method that would re-identify the information, but rather whether a reasonable person *in the position of the responsible party* would have foreseen a method that would re-identify the information.

Accordingly, the first interpretative avenue leads to the conclusion that the identifiability of data subjects must be determined in an objective, *context-specific* way.

### 2.2 The application clause and the research exception

The second interpretative avenue follows a different pathway through POPIA but reaches the same conclusion. POPIA’s application clause (Section 3) provides that POPIA applies only to *personal* information, which is information relating to an identifiable living natural person and, where it is applicable, an identifiable existing juristic person. Accordingly, in the health research context, the first question is whether the information *relates* to actual human research subjects (in other words, the information is not synthetic). And if the first question is answered in the affirmative, the second question is whether these human research subjects (i.e., the data subjects) are *identifiable* from the information. If the second question is also answered in the affirmative, POPIA applies to such information.

However, analogous to the position with the exclusions clause discussed above, it is not clear from the application clause *who* must not be able to *identify* the data subjects from the information: Nobody in the entire world, or the specific responsible party in possession of the information? Although this question is not explicitly answered in POPIA, the way in which the word “identifiable” is used elsewhere in POPIA, namely, in the research exception [Section 15 (3) (e)], does suggest the answer.

POPIA’s research exception allows for secondary research on personal information already collected without the need to re-consent the data subjects, but on condition that the responsible party ensures that the personal information used in such secondary research “will not be published in an identifiable form”. Thus, the data subjects must not be identifiable from the information *that is shared with the public*. Yet, there is no requirement in this section that the research institution must de-identify the personal information that is in its own possession—i.e., not shared with the public. This means that the research institution *itself* can retain its ability to identify the data subjects. Accordingly, POPIA contemplates identifiability to be determined from the perspective of the person or institution that is interacting with the relevant information. In other words, POPIA contemplates identifiability to be *context specific*.

Let me explain this from another angle: In the context of health research, POPIA’s research exception envisions the possibility of multiple versions of the same dataset. The dataset that contains personal information (call it “Dataset A”) can be used repeatedly for research purposes without the need to re-consent the data subjects. This can continue *ad infinitum*. Whenever an article is published based on the research, and whenever the underlying data must be provided as supplementary material to the journal, Dataset A or the relevant part of it that the article relies upon, must be de-identified (call it “Dataset A1”) before submitting it to the journal. This de-identification can be accomplished by either *deleting* all identifiable information in the derivative dataset or by *replacing* such information in the derivative dataset with a *pseudonym* that the public does not have access to. Whichever method is employed, the research institution complies with POPIA’s research exception, as the public (excluding the research institution) cannot identify the data subjects. Note that whether the derivative datasets are created by deleting information or by pseudonymisation makes no difference to the fact that the research institution remains in possession of Dataset A itself—the original dataset that contains all the personal information. Datasets A and A1 exist at the same time—one version of the dataset in “identifiable form,” another version *not* in “identifiable form”. This vision of what is entailed by POPIA’s research exception is clearly incompatible with *identifiable* meaning *identifiable by anyone in the world*, as the data subjects will indeed be identifiable by those with access to Dataset A.

In statutory interpretation, according to the principle of internal consistency, it is presumed that the meaning of a term used in a statute remains consistent throughout the statute (*Minister of the Interior v Machadodorp Investments Ltd*, 1957). Accordingly, identifiability should consistently be interpreted in a *context-specific* way.

### 2.3 Conclusion on interpretation

While POPIA does not overtly elaborate on pseudonymisation, POPIA’s provisions, when interpreted contextually and in light of established South African legal principles, lean towards an objective, context-specific understanding of data subject identifiability. Notably, the concept of “reasonably foreseeable method” intertwined with established legal precedents, and the contextual interpretation of the term “identifiability” in POPIA’s research exception, both converge on a perspective that grounds data subject identifiability in specific contexts. It is noteworthy that this interpretation aligns with the European position expressed in the *Single Resolution Board*.

## 3 POPIA’s application to pseudonymised datasets

In this section, I consider how the context-specific interpretation of identifiability in POPIA applies to pseudonymised datasets. First, I focus on the practical issue of determining whether a dataset is pseudonymised. I then consider the legal position under POPIA of each of the parties to a data transfer agreement, namely, the provider and the recipient, where the dataset that is transferred is pseudonymised.

### 3.1 Pseudonymisation in health research practice

When exactly is a dataset pseudonymised? In health research this question might not always have an obvious answer. Consider, for example, a research institution that conducts genomic research. It collects the data subjects’ names, phone numbers, gender, age group, race, and takes blood samples that are used to generate genomic data. All of these data are combined in a dataset. If the research institution replaces the data subjects’ names and phone numbers with unique codes, is the dataset pseudonymised? The answer depends on an assessment of whether the genomic data can identify a data subject. Say, for example, the research institution conducted genotyping (investigating the differences in individuals’ genotypes) and used a targeted approach of focusing only on specific portions of DNA instead of the entire genome. This targeted approach does not mean that the resulting data are not identifiable. In fact, genotyping data may contain unique genetic markers specific to an individual. This means that under the right circumstances or when combined with other datasets, an individual could potentially be identified. Although human whole-genome sequencing is relatively rare in South Africa, the same would obviously apply. On the other side of the spectrum, information on a single allele—even if rare—within a sufficiently large cohort would not be sufficient to identify a person.

If it is determined that the dataset still contains data that can identify a data subject, even after the data subjects’ traditional identifiers, such as their names and contact numbers have been replaced with codes, it means that the dataset has only been *partially* pseudonymised. Although this is a good data *security* measure (as it limits the risk of data subjects being identified), from a *legal* perspective it does not change the dataset’s status, as it remains inherently identifiable. In other words, for purposes of legal analysis, partial pseudonymisation is not pseudonymisation.

To build on the example above, an important question is whether a dataset that contains identifying genomic data can be pseudonymised? Similar to a dataset that contains high-resolution geolocation data that can be pseudonymised by—over and above replacing names with codes—lowering the resolution of the geolocation data to such an extent that such data can no longer identify any data subject, a dataset that contains genomic data can also be pseudonymised by lowering the dataset’s resolution in the sense that only broader, less granular data is retained. For example, exact genetic sequences can be replaced with information about whether a certain genetic marker is present or not. For certain datasets, it might be possible to generalise data by grouping them. However, depending on the kind of analysis that researchers intend to perform on the dataset, these techniques may entail sacrificing useful and valuable data, and their use is therefore not always appropriate or desirable.

In sum, therefore, a dataset is pseudonymised by taking the following steps: Allocating a unique code for each data subject; deleting all the traditional identifiers, such as name and phone number; where applicable, deleting any other identifying information, such as unique genetic markers specific to an individual, or changing such information to the extent that it can no longer identify any data subjects; and creating a dataset that links the unique codes of the data subjects with their identities, and keeping such linking dataset separate, confidential, and secure.

### 3.2 Transferring a pseudonymised dataset

Consider the following scenario: University X collects health information from research participants (data subjects). From the outset, University X employs a pseudonymisation system to ensure that the health information dataset that it is developing does not contain any identifying information of the data subjects. University X keeps the linking dataset separate, confidential and secure. The following legal questions are pertinent: First, does POPIA apply when University X processes its pseudonymised dataset? Second, if University X shares a copy of its pseudonymised dataset with University Y—but not the linking dataset—does POPIA apply when University Y processes the pseudonymised dataset?

### 3.3 The pseudonymised dataset in the hands of the provider

Although University X keeps the linking dataset secure, it possesses both the pseudonymised dataset and the linking dataset, and therefore has a reasonably foreseeable method at its disposal to re-identify the pseudonymised dataset. An important data safety measure for University X is having internal policies in place to ensure that the linking dataset is secured and that the researchers who are using the pseudonymised dataset do not have access to the linking dataset. But it does not change the fact that University X *qua* juristic person can re-identify the pseudonymised dataset.

Accordingly, in the hands of University X, the pseudonymised dataset constitutes *personal* information (information relating to *identifiable* living natural persons) and POPIA applies to such a pseudonymised dataset. This means that any processing of the information contained in the pseudonymised dataset by University X must be done in compliance with the relevant conditions for processing, as provided in POPIA. However, does such processing include transfer of the pseudonymised dataset to University Y? I return to this question after discussing University Y.

### 3.4 The pseudonymised dataset in the hands of the recipient

University Y possesses only the pseudonymised dataset and not the linking dataset, and therefore does not have a reasonably foreseeable method to re-identify the pseudonymised dataset. Accordingly, in the hands of University Y, the pseudonymised dataset does *not* constitute personal information and POPIA does *not* apply. It follows then that when University Y processes the information in the pseudonymised dataset, it is under no legal obligation to comply with any of POPIA’s conditions for processing.

### 3.5 Redux: transfer of the pseudonymised dataset by the provider

At the moment that University X transfers the pseudonymised dataset, the dataset is still personal information in its hands. This seems to suggest that University X must comply with POPIA’s rules regarding the transfer of the pseudonymised dataset to University Y. On the other hand, the act of transfer implies that the information will be placed in possession of the recipient. Common sense dictates that the act of transfer of information necessitates an orientation towards the *recipient*, instead of the provider.

This common-sense position can be strengthened by the following legal argument: South African law adheres to the doctrine of purposive interpretation (*Bertie Van Zyl Ltd v Minister for Safety and Security,* 2009). Thus, one should ask: What is the purpose of applying the rules of POPIA to the *transfer* of information? The purpose, I suggest, is to ensure that data subjects’ privacy rights are protected when the recipient receives the transferred information. This is why, for example, where the recipient is in a foreign country (see POPIA Section 72), it is legally relevant whether the *recipient* is subject to law, binding corporate rules or a binding agreement which provide an adequate level of protection for the processing of personal information. Would applying the rules of POPIA to a transfer where the transferred information will be non-personal information in the hands of the recipient ensure that data subjects’ privacy rights are protected when the transferred information is received by the recipient? The answer is clearly “no”. In the hands of the recipient the information is non-personal information. In other words, the recipient has no reasonably foreseeable method of identifying the data subjects and therefore their privacy rights are, from the outset, not at risk. It follows that when University X transfers the information in the pseudonymised dataset, it is under no legal obligation to comply with any of POPIA’s conditions for processing.

## 4 Conclusion

If my analysis is correct, namely, that identifiability in POPIA ought to be interpreted in a context-specific way, the transfer of pseudonymised datasets by providers and the subsequent processing of such datasets by recipients fall beyond POPIA’s scope of application. This result provides significantly more leeway for both providers and recipients of pseudonymised datasets. Does this leeway come at a cost for the privacy rights of data subjects? I suggest not. Nobody but the providers of the pseudonymised datasets—those who hold the key to re-identification of such datasets—have a reasonably foreseeable method of identifying the data subjects. And these providers remain bound by POPIA’s rules when they process pseudonymised datasets *within* their organisations, for example, when their own staff analyse the pseudonymised datasets for research purposes.

However, a note of caution is warranted. My argument hinges on the premise that a recipient does not possess reasonably foreseeable means to re-identify a (properly) pseudonymised dataset. Yet, scenarios can be imagined where this premise is challenged. For instance, if University X collected its research data in partnership with University Z, the latter might have the means to re-identify a pseudonymised dataset based on their joint research. However, many years later, staff members from University X might be oblivious to this past collaboration. Therefore, for practical reasons, I propose that the provider of a pseudonymised dataset should (a) internally examine the organisational history of the pseudonymised dataset and (b) query the recipient about any accessible information that could serve as a key to re-identify the dataset. Both (a) and (b) ought to be documented, with the results of (b) ideally being included in the parties’ data transfer agreement.

At the end of 2020, the Academy of Science of South Africa (ASSAf) embarked on a project to develop a Code of Conduct for Research (Code) in terms of POPIA. This project offers the opportunity to clarify when and how POPIA applies to pseudonymisation, and how pseudonymisation should be used in research. Academy of Science of South Africa (2023) recently submitted its proposed version of the Code to the South African Information Regulator for its consideration and eventual approval. The Information Regulator (2023) then published the proposed Code for public comment. The proposed Code defines pseudonymisation and embraces it as the default in all high-risk research. However, the proposed CCR does not address the essential issue of the relevance of context in the interpretation of identifiability. Given the widespread use and sharing of pseudonymised datasets in health research in South Africa, I suggest that the final Code should provide clarity on this highly consequential issue and illustrate its application to everyday research activities with practical examples. Moreover, since the use of pseudonymised datasets transcends the research milieu, the Information Regulator should publish a general guidance note to clarify this issue.

## Data Availability

The original contributions presented in the study are included in the article/supplementary material, further inquiries can be directed to the corresponding author.
